# Squamous Cell Carcinoma of the Thumb: Misdiagnosis and Consequences

**DOI:** 10.3390/jcm14134640

**Published:** 2025-06-30

**Authors:** Alessia Pagnotta, Luca Patanè, Carmine Zoccali, Juste Kaciulyte, Federico Lo Torto, Diego Ribuffo

**Affiliations:** 1 Hand and Microsurgery Unit, Jewish Hospital, 00186 Rome, Italy; pagale@me.com; 2 Plastic Surgery Unit, Department of Surgery, Sapienza University of Rome, 00185 Rome, Italy; 3 Orthopaedic Surgery Unit, Department of Surgery, Sapienza University of Rome, 00185 Rome, Italy; 4 UOC Chirurgia Oncologica della Mammella, Azienda Ospedaliero Universitaria Senese, 53100 Siena, Italy

**Keywords:** squamous cell carcinoma, thumb, hand tumour, diagnostic delay, misdiagnosis

## Abstract

**Background**: Cutaneous squamous cell carcinoma (SCC) is the most common primary malignant tumor of the hand, and its aggressive nature can lead to significant morbidity, particularly when affecting critical structures like the thumb. SCC in this location may arise in the periungual area or the pulp and frequently presents with non-specific symptoms such as swelling, nail deformity, or discharge, features that closely mimic common benign conditions. **Methods**: A retrospective study analyzed patients with neglected or misdiagnosed SCC of the thumb treated at the Hand and Microsurgery Unit of the Jewish Hospital, Rome, between 2015 and 2025. Patient demographics, duration from symptom onset to diagnosis, initial misdiagnoses, and imaging findings (X-rays, MRI, CT scans, lymph node sonography) were reviewed. Surgical interventions, histopathological grading, and postoperative management were documented, with long-term follow-up focusing on disease progression and patient survival. **Results**: Sixteen patients were included in the study. The mean age at surgery was 73.6 years (range: 55–93 years), with a mean delay of 8.2 months from symptom onset to diagnosis in 87.5% of cases. Initial misdiagnoses included verruca vulgaris, onychomycosis, paronychia, and osteomyelitis. Imaging consistently revealed soft tissue involvement, bony invasion, and occasional metastasis. Surgical approaches ranged from wide resection to amputation, with thumb reconstruction in selected cases and hand amputation in severe presentations. Long-term follow-up (mean 4.6 years) showed high morbidity, a reduction in hand function and QoL, and a 50% mortality rate, with two cases due to metastatic disease (12.5%). **Conclusions**: Thumb SCC presents diagnostic and therapeutic challenges, exacerbated by late diagnosis and initial misdiagnoses. Multidisciplinary management involving early recognition, comprehensive imaging, appropriate surgical interventions, and vigilant follow-up is crucial for optimizing outcomes.

## 1. Introduction

Squamous cell carcinoma (SCC) is the most common primary malignant tumor of the hand, accounting for the majority of non-melanoma skin cancers in this anatomically and functionally important region [1]. It arises from the malignant transformation of keratinocytes and is characterized by a variable degree of aggressiveness, depending on factors such as tumor differentiation, anatomical location, immune status, and time to diagnosis. While basal cell carcinoma (BCC) is more prevalent overall, SCC is disproportionately represented in the hand, with a reported BCC:SCC ratio of 1:14 in this region [2]. Unlike BCC, SCC has a well-recognized potential for local tissue invasion, bone involvement, and regional lymphatic metastasis, making early recognition and management essential for both oncologic and functional outcomes [3]. The progression of SCC typically follows a continuum from precursor lesions such as actinic keratosis, characterized by localized epidermal atypia, to carcinoma in situ (Bowen’s disease), where full-thickness keratinocyte atypia extends to the basal layer without invading the basement membrane. From these atypical foci, invasive SCC develops, characterized by the infiltration of these abnormal cells beyond the basement membrane. Histologically, invasive SCC is classified into well, moderately, and poorly differentiated forms based on the degree of nuclear atypia, keratinization patterns, and frequency of mitoses, which influence its clinical behavior and prognosis [4].

The development of SCC is intricately linked to various environmental and genetic factors. Chronic exposure to ultraviolet (UV) radiation [3], both occupational and from environmental sources, remains the predominant risk factor, particularly affecting sun-exposed areas such as the dorsal surfaces of the hands. Other carcinogenic agents include arsenic, polycyclic hydrocarbons [5], and infections with human papillomavirus (HPV) [6], which have been implicated in SCC pathogenesis.

Additionally, individuals with genodermatoses like xeroderma pigmentosum and Huriez syndrome [7,8] and those with chronically inflamed or injured skin (e.g., burns, ulcers, and cutaneous manifestations of lupus and lichenoid diseases) exhibit an increased susceptibility to SCC development.

Huriez syndrome, also known as sclerotylosis, is a rare autosomal dominant genodermatosis characterized by congenital scleroatrophy of the distal extremities, palmoplantar keratoderma, and nail hypoplasia [9].

Despite its prevalence and association with UV radiation, SCC on the hand, particularly in elderly patients, often presents diagnostic challenges. When SCC arises in critical hand structures such as the thumb, diagnosis and treatment are often complicated by atypical presentation and functional concerns. The thumb plays a fundamental role in opposition, prehension, and overall hand function. SCC in this location may arise in the periungual area or the pulp and frequently presents with non-specific symptoms such as swelling, nail deformity, or discharge—features that closely mimic common benign conditions, including paronychia, onychomycosis, pyogenic granuloma, and verruca vulgaris. Lesions affecting the nail unit or those associated with a history of trauma can be easily overlooked or misdiagnosed by dermatologists and surgeons.

These similarities often lead to initial misdiagnosis, resulting in delayed referral, a lack of biopsy, and inappropriate conservative treatment, all of which may allow progression to deeper tissue layers or adjacent bone. In such cases, the potential for extensive soft tissue involvement and bony infiltration may necessitate aggressive interventions like wide local excision, amputation, or complex reconstructions [10,11], impacting both functional outcomes and quality of life for patients.

The balance between oncological safety and functional preservation in thumb tumors presents a complex decision-making scenario, particularly in elderly patients with limited surgical tolerance or high comorbidity burden.

This study aims to retrospectively analyze the clinical presentation, diagnostic challenges, and management outcomes of neglected or misdiagnosed squamous cell carcinoma (SCC) of the thumb. Specific objectives include characterizing the demographic profile and initial misdiagnoses, evaluating the extent of disease using imaging modalities, and assessing the histopathological features and treatment strategies employed, focusing on functional outcomes and patient survival over long-term follow-up.

## 2. Materials and Methods

### 2.1. Study Design and Setting

This retrospective study included patients treated for squamous cell carcinoma (SCC) of the thumb at the Hand and Microsurgery Unit of the Jewish Hospital, Rome, between January 2015 and March 2025. All patients had histologically confirmed SCC of the thumb and presented with either an initial misdiagnosis or a significant diagnostic delay. The hospital is a tertiary referral center with subspecialty expertise in hand oncology and reconstructive surgery.

### 2.2. Inclusion and Exclusion Criteria

Patients were eligible for inclusion if they met the following criteria: age ≥ 18 years; primary SCC of the thumb, confirmed by incisional biopsy; initial misdiagnosis or a diagnostic delay from symptom onset to diagnosis; complete medical records and a minimum follow-up of 6 months.

Patients were excluded if they had any of the following: SCC involving other fingers without thumb involvement; SCC secondary to metastasis from another primary site; incomplete data or loss to follow-up before definitive treatment.

### 2.3. Data Collection

Patient demographics, including age at surgery and duration from symptom onset to diagnosis, were recorded.

Initial misdiagnoses were accurately recorded by the first author during initial consultation. Each patient underwent comprehensive imaging studies, consisting of X-rays and MRI of the hands and wrists, to investigate soft tissue involvement, bony invasion, and pathologic fractures of the thumb, and CT body scans and axillary lymph node sonography, to evaluate the presence of metastasis.

An incisional biopsy was performed in all cases. Histopathological analysis graded the lesions based on the percentage of undifferentiated cells: G0 (in situ carcinoma), G1 (well-differentiated, <25% undifferentiated cells), G2 (moderately differentiated, <50% undifferentiated cells), and G3 (poorly differentiated, >75% undifferentiated cells) [4].

Surgical procedure type, presence of nodal metastasis, and reconstructive approach (if performed) were also analyzed.

In our study, we implemented the QuickDASH score to assess hand functionality following surgery and to evaluate patients’ quality of life. This validated tool allowed us to objectively measure the impact of surgical interventions on hand mobility, pain levels, and daily functional activities. By incorporating the QuickDASH score, we were able to gain valuable insights into the functional outcomes and overall well-being of patients after treatment for squamous cell carcinoma of the thumb.

The QuickDASH (Disabilities of the Arm, Shoulder, and Hand) score is a shortened version of the original DASH questionnaire, designed to assess the functional status and symptoms of the upper limb. It consists of 11 items that evaluate pain, physical function, and the impact of upper limb disability on daily activities. The QuickDASH provides a comprehensive measure of hand function, allowing clinicians to assess the severity of impairment and monitor changes over time, making it a valuable tool in evaluating outcomes after surgery or treatment for upper limb conditions.

### 2.4. Imaging and Staging

All patients underwent preoperative imaging including the following: plain radiographs (X-rays) of the hand to evaluate bone involvement; Magnetic Resonance Imaging (MRI) to assess soft tissue extension and tendon, joint, or neurovascular involvement (performed with 1.5 Tesla scanner (Tesla, Erlangen, Germany) using T1, T2, STIR, and post-contrast sequences); Contrast-enhanced Computed Tomography (CT) of the chest, abdomen, and pelvis for staging in suspected advanced cases; axillary and epitrochlear lymph node ultrasound in all cases.

### 2.5. Surgical Management

Treatment decisions were based on clinical and imaging findings, with the goal of achieving negative margins while preserving function when feasible. Surgical approaches included the following: wide local excision (when bone involvement was excluded); partial amputation at the distal or proximal phalanx; metacarpal or transmetacarpal hand amputation in cases with extensive invasion; thumb reconstruction using local flaps in selected patients, based on age, comorbidities, and extent of resection.

Histological margins and tumor grade were confirmed in all excised specimens.

### 2.6. Follow-Up and Outcome Measures

Postoperative follow-up was conducted by a multidisciplinary team of surgeons, dermatologists, and oncologists. Follow-up visits occurred every 3–6 months during the first 2 years, then annually. Evaluations included the following: physical examination; imaging when indicated (especially in high-grade or advanced tumors); oncologic status (recurrence, metastasis); overall survival.

## 3. Results

Sixteen patients (13 males, 3 females) with neglected or clinical misdiagnosed SCC of the thumb operated on by the first author (A.P.) were included in the study (Table 1 and Table 2). The mean age at surgery was 73.6 years (range: 55–93 years), with a mean duration from symptom onset to diagnosis of 8.2 months (range: 3–22 months). Fourteen patients (87.5%) received a cancer diagnosis more than 6 months after symptom onset.

### 3.1. Initial Misdiagnosis

All patients had received at least one initial clinical misdiagnosis prior to referral. The most frequent were as follows:

- Paronychia: six patients (37.5%);
- Onychomycosis: three patients (18.7%);
- Verruca vulgaris: three patients (18.7%);
- Osteomyelitis or soft tissue infection: four patients (25%).

### 3.2. Imaging Findings

All patients underwent plain radiographs and MRI of the hand and wrist. Imaging consistently revealed the following:

- Soft tissue involvement in all 16 patients (100%);
- Bone invasion or pathologic fracture in all cases (100%);
- Suspicious lymph nodes on axillary/epitrochlear ultrasound in 2 cases (12.5%);
- Confirmed nodal metastasis in 1 patient (6.25%) following biopsy.

CT scans of the chest and abdomen were performed preoperatively in all cases. No distant metastases were found at diagnosis.

### 3.3. Histopathological Grading

Based on histological analysis, the following data were obtained:

- Twelve patients (75%) had grade 1 (well-differentiated) SCC;
- Four patients (25%) had grade 2 (moderately differentiated) SCC;
- No patients had grade 3 (poorly differentiated) or in situ carcinoma.

All tumors were confirmed with incisional biopsy prior to surgery. In two patients with clinical lymphadenopathy, sentinel lymph node biopsy was performed; one was positive for metastasis.

### 3.4. Surgical Interventions

**Figure 1:**
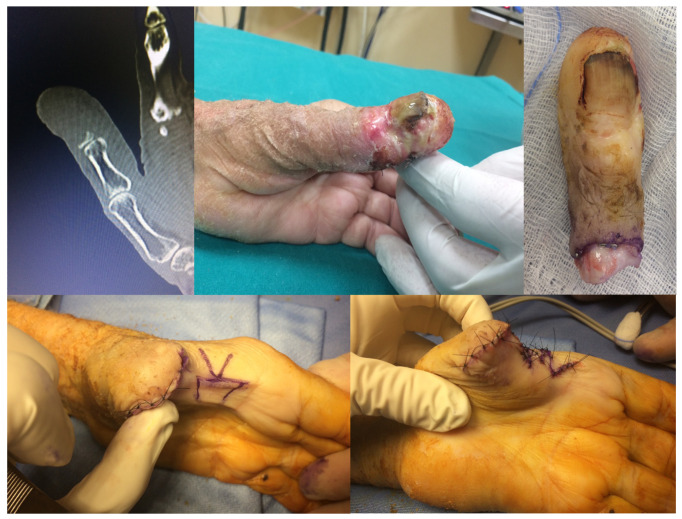
Clinical Case 1: Complete osteolysis of the distal thumb phalanx. Pre-operative findings revealed SCC originating from the periungual region, initially misdiagnosed as a paronychia, with subsequent extension to the skin of the proximal phalanx. Operative management included M1 amputation and Z-plasty reconstruction of the first web space.

**Figure 2:**
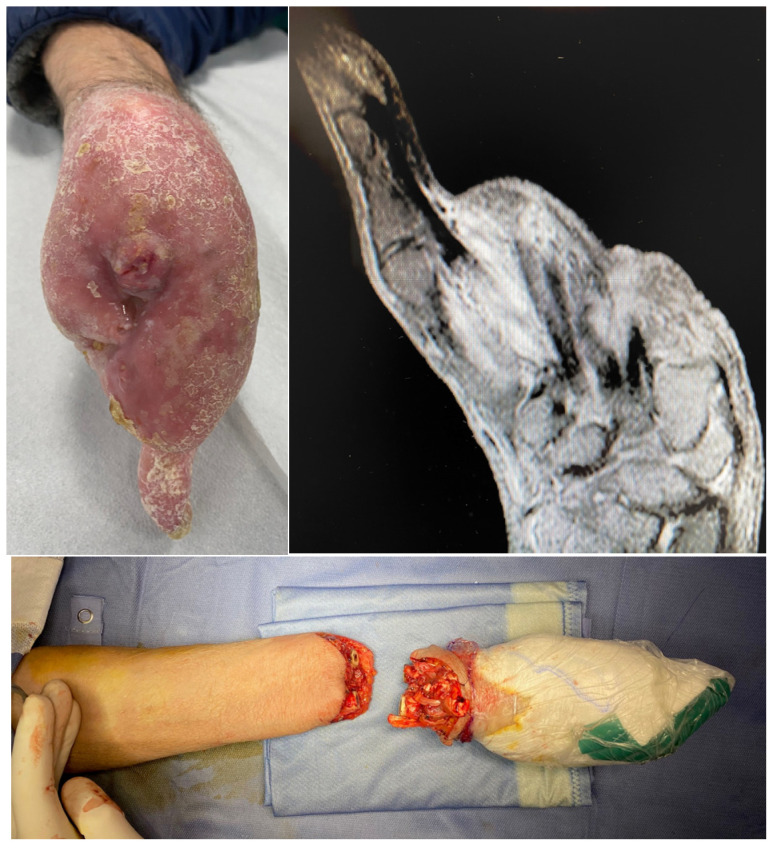
Clinical case 2: Patient with Huriez syndrome presenting with recurrent digital tumors, initially misdiagnosed multiple times. Persistent infiltration of the thumb necessitated hand amputation performed by the author at the forearm level.

Surgical treatment included the following (Figure 1 and Figure 2):

- Wide local excision in four cases (25%);
- Amputation at the proximal phalanx (P1 level) in six cases (37.5%);
- Amputation at the first metacarpal (M1 level) in three cases (18.7%);
- Hand amputation in three cases (18.7%).

**Figure 3:**
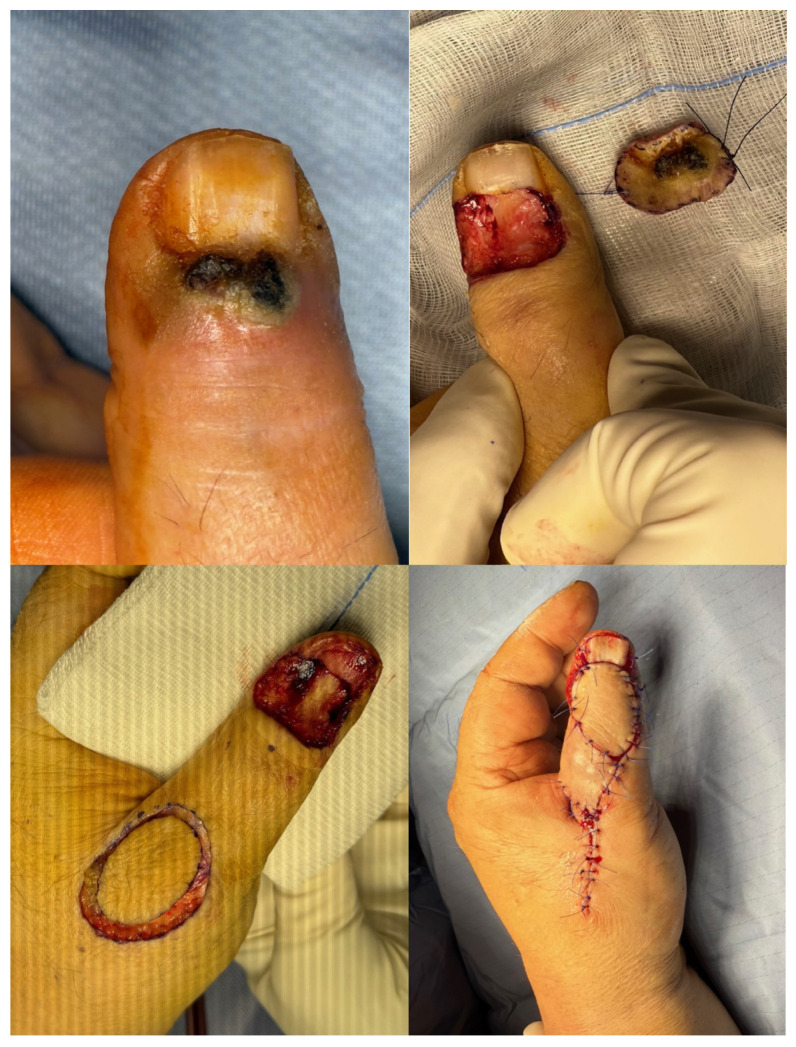
Clinical case 3: Biopsy confirmed in situ tumor, definitive grade 1. Wide resection of the nail bed and matrix down to the skeletal plane. Reconstruction using ulnar dorsal thumb flap following initial misdiagnoses of paronychia and wart.

Thumb reconstruction was performed in four patients (25%) using local flaps (Figure 3). Reconstruction was more commonly offered to younger or functionally active patients. Patients who underwent distal amputation often declined reconstruction. Hand amputation was performed in cases with extensive local invasion or in the context of severe underlying conditions, such as Huriez syndrome. 

### 3.5. Follow-Up and Outcomes

Follow-up was performed by oncologists and surgeons, with a minimum time of 6 months to a maximum of 9 years. The mean follow-up time from treatment was 4.6 years.

At the latest follow-up, the average QuickDASH score for the 16 patients was 48.5, with a standard deviation of 25.3. The scores ranged from 0 (no disability) to 84 (severe disability), indicating a wide variability in post-surgery functional outcomes. While some patients retained nearly full hand function, others experienced significant impairment.

During follow-up, 8 out of 16 patients (50%) were deceased, with metastatic disease identified as the cause in 2 cases. In the remaining patients, no clinical or radiological (RMN) signs of recurrence were present at follow-up.

## 4. Discussion

The thumb, being the most affected digit by SCC and crucial for hand function, presents unique challenges in both diagnosis and treatment, especially in the elderly population. Age-related anatomical changes in joints, muscles, tendons, bones, nerves, fingernails, and skin, coupled with neural control alterations, significantly impact hand function and dexterity. Oncological resections of the thumb due to SCC further exacerbate these challenges, often limiting autonomy and mobility. For example, patients reliant on walking aids would experience a significant loss of independence if they were unable to use their thumbs effectively.

For small, low-risk lesions of the hand SCC, various treatment modalities such as shave excision, curettage and cautery, cryotherapy, Mohs micrographic surgery, and electrochemotherapy have been employed to preserve thumb function [12,13]. These approaches aim to balance oncological control with functional outcomes, offering hope for salvaging hand function in less aggressive cases.

However, preserving thumb function becomes exceedingly challenging in cases of locally aggressive SCC necessitating wide local excisions or even amputations, similar to what is needed for other soft tissue tumors of the hand [14,15,16]. Rates of recurrence and metastasis for SCCs affecting the hand are notably higher compared to those of general cutaneous SCC [17]. This heightened propensity is attributed to the anatomical complexity and functional importance of the hand, where SCCs can infiltrate critical structures such as tendons, joints, and nerves, increasing the likelihood of residual disease and metastatic spread. The potential for metastasis to regional lymph nodes and distant organs underscores the criticality of timely diagnosis and intervention [18,19]. Our findings highlight the varied delays in diagnosing thumb SCC, ranging from 3 to 22 months, with misdiagnoses like paronychia and verruca vulgaris contributing significantly to diagnostic challenges.

In our series, the shortest delay in diagnosis was observed in a patient presenting with a thumb pulp lesion initially mistaken for a verruca, while the longest delay occurred in a case where SCC was misdiagnosed as osteomyelitis with pathological fractures. SCCs affecting the nail unit are commonly misdiagnosed as verruca vulgaris, pyogenic granuloma, onychomycosis, or paronychia due to the ambiguous nature of nail pathology. Delays in diagnosing nail unit SCCs are frequently encountered rather than exceptional.

At our hospital, which specializes in hand pathology, prompt diagnosis is crucial to mitigate the spread of SCC into deep soft tissue structures, which can involve critical anatomical components such as extensor and flexor tendons, vascular and nervous bundles, joints, and bone. For instance, we suggest that every suspicious lesion must be biopsied and, in case of confirmed diagnosis of cutaneous SCC, proper imaging must be carried out to plan an adequate surgical resection. Delayed diagnoses not only complicate treatment but also compromise hand dexterity and prehension following demolitive surgery, emphasizing the importance of early recognition and management of thumb SCC [17,18,19,20,21,22,23,24,25,26].

The QuickDASH score is a comprehensive tool used to assess the ability to perform various manual activities, but it is not specific to the operated hand [27,28]. For example, a patient who has undergone a hand amputation may still perform tasks, such as washing, with the remaining hand, and thus may not experience total disability. However, in elderly patients who are already highly limited due to age-related factors, the global disability can be more pronounced. In our study, we implemented the QuickDASH score to evaluate residual hand function and the overall quality of life (QoL) following surgery. The score ranges from 0, which indicates no disability, to 100, which reflects the most severe disability.

The results of our investigation revealed that most patients experienced significant disability post-surgery, with many scoring high on the QuickDASH scale, indicating substantial impairment in hand function. This underscores the critical importance of early detection and prompt, radical surgical intervention in managing squamous cell carcinoma of the thumb. Timely treatment is essential to preserving hand function and improving QoL, as delays or incomplete resections can result in greater functional loss and a more severe impact on daily activities. Early and effective management not only reduces the risk of recurrence but also helps maintain the patient’s independence and functional capacity, ultimately improving long-term outcomes.

Interestingly, when analyzing the data, the type of surgery performed was not found to be correlated with the QuickDASH scores. This suggests that while surgical intervention is crucial, factors such as the timing of surgery, tumor progression, and patient-specific factors (e.g., age, comorbidities) may have a greater impact on functional outcomes than the surgical approach itself.

Our study is the first study that provides evidence that underscores the clinical and pathological characteristics of SCC affecting the thumb, revealing a predominance in elderly patients with significant delays in diagnosis. The majority of patients presented with well to moderately differentiated SCC, necessitating a spectrum of surgical interventions from wide local excisions to amputations. Despite efforts to preserve thumb function through reconstructive techniques, significant post-operative morbidity and mortality, primarily from metastatic disease, highlight the complex interplay between oncological imperatives and functional outcomes.

## 5. Future Directions

These findings underscore the complex interplay between oncological imperatives and functional outcomes in managing thumb SCC. Improving diagnostic vigilance and patient education are critical to enhancing early detection and management of thumb SCC. Multidisciplinary collaboration among dermatologists, plastic surgeons, and oncologists is essential for optimizing treatment strategies and improving patient outcomes. Future research should focus on refining diagnostic protocols and exploring novel therapeutic avenues to mitigate the morbidity associated with advanced SCC. In the case of thumb SCC, Artificial Intelligence (AI) applications could assist in distinguishing between benign and malignant lesions, enabling clinicians to make quicker, more accurate decisions. Furthermore, machine learning (ML) models trained on large datasets of dermatologic images could improve the precision of diagnoses, especially in challenging cases like nail unit SCC, which are often misdiagnosed, by implementing a diagnostic tool in everyday practice [29].

## 6. Conclusions

Early recognition and prompt management of SCC, particularly in at-risk populations, are critical. It is imperative to prioritize comprehensive patient education, regular skin examinations, and timely biopsy of suspicious lesions to facilitate early detection and effective treatment planning. Proactive management strategies can significantly mitigate the morbidity associated with advanced SCC, thereby improving patient prognosis and enhancing overall quality of life.

## Figures and Tables

**Table 1:** Patient demographics and characteristics.

Patients Included (n)	16
Gender (male)	13
Age at surgery (years, range)	73.6 (55–93)
Duration from symptom onset to diagnosis (months, range)	8.2 (3–22)
Patients diagnosed >6 months after onset (n, %)	14 (87.5)
Initial misdiagnoses (n, %)	
Verruca vulgaris	3 (18.7)
Onychomycosis	3 (18.7)
Paronychia	6 (37.8)
Osteomyelitis/infection	4 (25)
Metastatic disease at diagnosis (n, %)	1 (5.8)
Histopathological grading (n, %)	
Well-differentiated SCC (grade 1)	12 (75)
Moderately differentiated SCC (grade 2)	4 (25)
Surgical interventions (n,%)	
Wide resection	4 (25)
Amputation (P1 level)	6 (37.6)
Amputation (M1 level)	3 (18.7)
Hand amputation	3 (18.7)
Thumb reconstruction (n, %)	4 (25)
Local flap	4 (25)
Follow-up (years, range)	4.6 (0.5–9)
Patient mortality during follow-up (n, %)	8 (50)
Metastatic disease	2 (12.5)

**Table 2:** Detailed patient records.

Patient	Age	Misdiagnosis	Surgery	Grading	QuickDASH
1	93	Paronychia	P1 amputation	G1	84
2	74	Verruca vulgaris	P1 amputation	G2	36
3	58	Ostemyelitis	M1 amputation	G1	52
4	76	Traumatic paronychia	P1 amputation	G1	47
5	75	Verruca vulgaris	P1 amputation + thumb soft tissue resection	G1	40
6	59	Huriez, infection	Hand amputation	G1	72
7	87	Paronychia	Wide resection	G2	68
8	75	Infection	Hand amputation	G2	75
9	55	Onychomycosis	Wide resection	G1	0
10	60	Paronychia	Wide resection	G1	9
11	80	Infection	Hand amputation	G2	77
12	75	Onychomycosis	M1 amputation	G1	47
13	68	Onychomycosis	Wide resection	G1	2
14	88	Verruca vulgaris	P1 amputation	G1	61
15	72	Paronychia	M1 amputation	G1	54
16	63	Paronychia	P1 amputation	G1	52

## Data Availability

The original contributions presented in this study are included in the article. Further inquiries can be directed to the corresponding author.
